# Flickering Stimuli Do Not Reliably Induce Visual Hallucinations in Parkinson’s Disease

**DOI:** 10.3233/JPD-191635

**Published:** 2019-07-30

**Authors:** Angeliki Zarkali, Andrew J. Lees, Rimona S. Weil

**Affiliations:** a Dementia Research Centre, University College London, London, UK; b Reta Lila Weston Institute, University College London, London, UK; c Wellcome Centre for Human Neuroimaging, University College London, London, UK

**Keywords:** Parkinson’s disease, visual hallucinations, stroboscopic light, hallucination state

## Abstract

Visual hallucinations are a common and often distressing feature of Parkinson’s disease; they are ephemeral and capricious, making them difficult to study but tend to be more prominent in dim illumination. Flickering stimuli can induce simple hallucinations even in healthy individuals. We tested a stroboscope and an equivalent full-screen flickering stimulus in 16 participants: 7 patients with Parkinson’s and habitual visual hallucinations, 6 Parkinson’s patients without hallucinations and 3 controls. Both flicker sources induced varied geometrical hallucinations in 4 participants (25%) and complex hallucinations in 1 but neither induced typical Parkinson’s-associated hallucinations.

## INTRODUCTION

Visual hallucinations are a frequent and often distressing symptom of Parkinson’s disease (PD) affecting 30–70% of patients [1, 2]. The phenomenology of PD-associated hallucinations is rich and varied, including both complex formed visual imagery in the absence of a stimulus (the classical definition of hallucinations [3]), and other hallucinatory phenomena such as less formed passage hallucinations, misperceptions and illusions [1, 2–4]. When phantasmagorical they can sometimes be distressing and frightening and have been associated with increased mortality [5], higher likelihood of nursing home placement [6], worse quality of life [7] and increased carer burden [6].

A significant impediment in studying PD hallucinations is their unpredictable occurrence. Visual hallucinations can be induced, even in healthy volunteers using a stroboscope, or by viewing a flickering stimulus [1, 2]. Although reported flicker-induced hallucinations are simple hallucinations, taking the form of geometrical shapes or colours, they could be a useful surrogate marker for the simpler hallucinatory phenomena that patients with PD experience which can be triggered or preceded by a percept [4]. We reasoned that flicker might be a useful “stress-test”, that could be used in routine clinical settings to determine whether a patient with PD was at risk of visual hallucinations or as a tool to induce hallucinations in experimental conditions.

## METHODS

### Flickering stimuli

**Fig.1:**
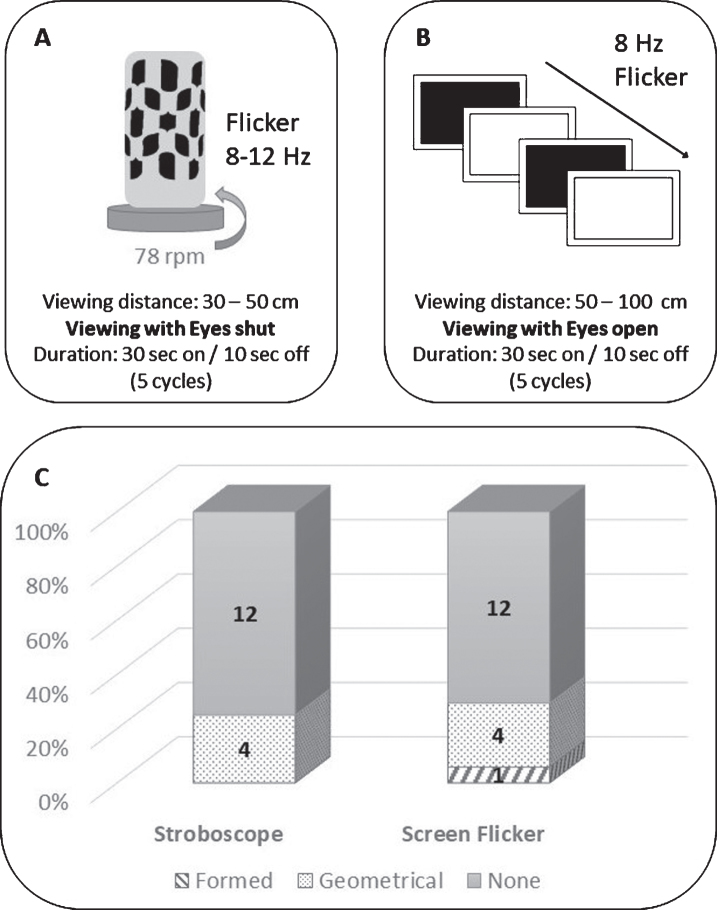
A. Illustration of the Stroboscope: Lightbulb encased in cardboard case with cutout holes (black), placed on top of turntable rotating at 78 rpm. Produces a flickering stimulus with a frequency between 8–12 Hz. Viewed with eyes shut. B. Illustration of the Full-screen flicker: Alternating fully black and fully white screen with a frequency of 8 Hz. Viewed with eyes open. C. Induced hallucinations: Blue: formed hallucinations, Orange: Geometrical Hallucinations, Grey: No hallucinations.

Two stimuli were studied: a stroboscope (identical to the Dreamachine used by the artist Brion Gysin and the writer William S Burroughs) and a full-screen computer flicker stimulus [8]. The stroboscope consisted of a lightbulb encased in cardboard with cut-out holes, as stated in Brion Gysin’s ‘Dreamachine Plans’. The lightbulb and card casing are secured upon a turntable which rotates at 78 rpm to produce light flicker at a frequency between 8 and 12 Hz (Fig. 1A) [8]. Participants viewed the stroboscope at a 30 cm distance; they faced the lightbulb with their eyelids shut. The stroboscope was controlled by a script on MATLAB R2014a (Mathworks Inc., 2014); on time of 30 s and off for 10 s per cycle for a total of 5 cycles.

Additionally, a full screen flicker stimulus was presented on a Dell latitude 3340 laptop screen (Dell (Round Rock, Texas, USA)); the stimulus was generated using MATLAB version R2014a and Cogent (http://www.vislab.ucl.ac.uk/cogent_2000.php). The stimulus consisted of an alternating fully black and fully white screen at a frequency of 8 Hz; full screen presentation was chosen to achieve maximum contrast (Fig. 1B). Frequency of 8 Hz flicker was chosen as this falls within the reported effective range (5–30 Hz) of flicker in inducing hallucinations and was proven to be most effective during pilot testing of different frequency ranges [9]. Patients were instructed to sit at a distance of 50 cm from the laptop screen and keep focus on the screen with their eyes open. The stimulus was presented for a total of 5 cycles: on time of 30 s and an off time of 10 s per cycle.

Both stimuli were observed in a dark room. Participants were instructed to indicate the onset of hallucinations, with qualitative descriptions collected during debriefing.

### Study participants

All patients fulfilled the Queen Square Brain Bank criteria for the diagnosis of PD [10]. Patients were divided into habitual hallucinators (VH) (7 patients) if they scored≥1 on question two of the Movement Disorder Society Unified Parkinson’s Disease Rating Scale (UPDRS: “Over the past week have you seen, heard, smelled or felt things that were not really there?”) [11] and non-hallucinators (6 patients). Three healthy controls were also tested.

### Clinical assessments

Further qualitative and quantitative details on the experienced hallucinatory phenomena were collected with the University of Miami Parkinson’s Disease Hallucinations Questionnaire (UM-PDHQ) [12]. Assessments of motor function included the Hoehn & Yahr and the UPDRS [11]. The Mini-Mental State Examination (MMSE) and Montreal Cognitive Assessment (MoCA) were used as measures of general cognition [13]. Visual acuity was assessed using the 6-meter Snellen chart. All participants had corrected bilateral acuity of > = 6/6. Contrast sensitivity was measured using thePelli-Robson test [14]. Visuospatial performance was measured using Benton’s Judgment of Line [15], Visual Object and Space Perception Battery [16] and the Hooper Visual Organization Test [17]. The Hospital Anxiety and Depression Scale (HADS) was used to assess mood [18] and the REM Sleep Behaviour Disorder (RBDSQ) to assess sleep [19]. All assessments and the experimental task were performed with patients in the ON state.

## RESULTS

The patients with and without hallucinations did not differ in age (*p* = 0.443), disease duration (*p* = 0.191), MMSE (*p* = 0.327), MOCA (*p* = 0.172), visual acuity (*p* = 0.301), or contrast sensitivity (*p* = 0.138) but the two control patients were younger than the patients with PD (*p* < 0.001) Patients with hallucinations had higher UPDRS total score (*p* = 0.037), reflecting non-motor symptom burden in this population.

Recruitment in our study was impeded by hesitation of patients to take part, for fear of inducing permanent hallucinations or epileptic seizures. Indeed of 26 invited participants (all taking part in other study on hallucinations) only 16 consented to take part. Despite these concerns, on follow up communication, two weeks after participation, we found no adverse effects of the flickering stimuli.

The demographics and clinical assessments in patients with PD in our cohort are seen in Table 1.

**Table 1:** Study group characteristics

Attribute		PD VH *n* = 7	PD non VH *n* = 6	*p* value
Demographics	Age (y)	70.6 (10.1)	72.2 (5.3)	0.443
	Years in Education	15.7 (1.4)	16.0 (1.8)	0.281
Mood (HADS)	Depression score	4.8 (1.8)	0.5 (1)	0.109
	Anxiety score	3.7 (2.6)	1.5 (1.9)	0.385
Vision	Visual acuity (bilateral)	0.92 (0.2)	0.96 (0.2)	0.301
	Pelli Robson (bilateral)	1.5 (0.2)	1.7 (0.1)	0.138
Neuropsychology	MMSE	27.6 (2.8)	29.2 (0.8)	0.327
	MOCA	27.2 (4.1)	25 (1.7)	0.172
Attention	Digit span backwards	7.7 (2.7)	5.8 (1.7)	0.414
	Stroop: Colour (sec)	38.9 (16.7)	46.4 (24.3)	0.360
Executive function	Stroop: Interference (sec)	82.1 (32.1)	60.2 (13.9)	0.260
	Category fluency	19.3 (5.4)	20.3 (1.9)	0.333
Memory	Word Recognition Task	23.6 (1.6)	23.3 (1.8)	0.384
	Logical Memory (delayed)	13 (3.1)	10 (4)	0.215
Language	Graded Naming Task	21.2 (5)	24 (3.5)	0.214
	Letter Fluency	12.4 (5.5)	13.8 (4.8)	0.124
Visuospatial	VOSP	52.5 (4.6)	55.3 (2.3)	0.281
	Benton’s Judgement of Line orientation	20.2 (7.9)	24.3 (4.2)	0.331
	Hooper	19.3 (7.8)	24.6 (3.1)	0.141
Disease specific	UPDRS	61.7 (25.4)	40.7 (17.8)	0.039
	LEDD (mg)	317.1 (134.9)	384.9 (250.6)	0.192
	Disease duration	4.9 (3)	3.5 (1.8)	0.191
	RBDSQ	3.1 (2.3)	4 (1.8)	0.156

PD VH: Patients with Parkinson’s disease and visual hallucinations; PD non VH: patients without visual hallucinations All data shown are mean (SD); HADS: Hospital anxiety and depression scale; MMSE: Mini-mental state examination; MOCA: Montreal cognitive assessment; UPDRS: Unified Parkinson’s disease rating scale; LEDD: Total Levodopa equivalent dose; RBDSQ: REM sleep behaviour disorder screening questionnaire.

### Stroboscope stimulation induced varied geometrical hallucinations

Fourteen of 16 participants (87.5%) were able to tolerate 10 cycles of each stimulus; the remaining two tested 5 cycles of each stimulus. The stroboscope induced visual hallucinations in only 4 of 16 participants; Two participants reported coloured hallucinations: one participant experienced moving blue dots, the other static neon pink and blue dots. The remaining two participants who experienced geometrical hallucinations with the stroboscope, reported white and black patterns; these also varied, including bars, squares and patterns reminiscent of “refractive diamonds”.

### Full screen flicker induced complex hallucination in only one participant

The full screen flicker stimulus also induced hallucinations in 4 of 16 participants; two of whom had also experienced geometric hallucinations with the stroboscope. The phenotype of these was more consistent than the stroboscopic induced hallucinations, including mainly a black and white grid pattern and a “spinning tunnel” of white light. Only one participant experienced formed hallucinations which he described as “a set of squares arranged on top of each other with circles in the middle” which resembled a filling cabinet.

No participants experienced typical formed hallucinations of people or animals that characterise PD-associated hallucinations [1, 20].

### Induced hallucinations not reflecting hallucination trait

The rate of induced hallucinations did not differ between PD patients with and without hallucinations either on the stroboscope or the full screen flicker (*p* = 0.247): geometrical hallucinations were experienced by 3 PD/VH patients (42.8%), 2 PD non VH (30%) and 1 control (33.3%). Of the 7 participants with PD who were habitual hallucinators, none experienced their habitual hallucinations or complex hallucinations with flicker. The one participant who experienced complex hallucinations with the flickering stimulus was not a habitual hallucinator and did not differ from the rest of the group in motor, visual or cognitive assessments.

## DISCUSSION

We have shown that a flickering stimulus, both using the classical stroboscope and an equivalent full screen flicker can induce geometrical hallucinations in some patients with PD. This is in keeping with past research with flicker-induced hallucinations being reported as early as 1819 [9, 21, 22].

A flickering stimulus has previously been used to study brain activation during induced geometrical hallucinations in PD [23]. In that study a 3 Hz full screen stimulus was used during fMRI; comparisons of interest were stroboscopic vs no visual stimulus in PD patients with and without hallucinations and revealed greater frontal and subcortical activation and less visual cortical activation in hallucinating compared with non-hallucinating patients [23]. The authors recognized that generalization of their study results was limited by the fact that none of the participants experienced their habitual hallucinations during scanning. The stimulus frequency was low (3 Hz) and the total duration was also short (6 min) which could account for the stimulus failure to induce hallucinations.

In our cohort, using a higher frequency and longer stimulus exposure, both the stroboscope and the full screen flickering stimulus also failed to consistently reproduce complex hallucinations. Whilst difference frequencies have been shown to differ in their effectiveness of inducing hallucinations, we used a high frequency within the most effective range [22, 24]; based on our own pilot testing.

Viewing distance could be a limiting factor to the stimuli success; according to the inverse square law of light, as distance increases from a point source (the lightbulb in our stroboscope), light intensity decreases. A high light intensity may be crucial as the stroboscope is viewed with closed eyelids and to achieve adequate retina stimulation strong illumination of the eyelids is required. This was not an impediment in the full-screen flicker and indeed this stimulus was slightly more effective in inducing stereotypical geometrical hallucinations.

It is also interesting to note that visual hallucinations tend to occur in low stimulus environments, and especially in people’s own homes [1]. In this way, any test situation, which is likely to be more stimulating, will also reduce the likelihood of hallucinations. This may have further reduced the threshold for triggering hallucinations in our study.

We studied the efficacy of flickering stimuli in a small cohort of patients with PD and controls. Although inferences are limited by the small number of participants, we found that less than 1 in 3 participants experienced flicker-induced hallucinations; these were almost uniformly geometrical in phenomenology and were not more prominently reported by those with habitual hallucinations. Further testing in a larger cohort, given the lack of signal in our study and the possible side effects from flicker exposure, such as migraine, would not be beneficial.

Whilst our study does not provide mechanistic insights into visual hallucinations, our findings flickering stimuli, including the stroboscope are, not a reliable and predictable stress-test to induce visual hallucinations in PD.

## FUNDING

AZ is supported by an Alzheimer’s Research UK Clinical Research Fellowship (2018B-001). RSW is supported by a Wellcome Clinical Research Career Development Fellowship (201567/Z/16/Z).

## CONFLICT OF INTEREST

The authors have no conflict of interest to report.
